# Disseminated Cryptococcosis revealed by transverse myelitis in Immunocompetent patient: a case report and review of the literature

**DOI:** 10.1186/s12883-020-1598-6

**Published:** 2020-01-10

**Authors:** Fangfang Qu, Zhenzhen Qu, Yingqian Lv, Bo Song, Bailin Wu

**Affiliations:** 1 Department of Respiratory and Critical Care Medicine, Institute of Respiratory Diseases, the Second Hospital of Hebei Medical University, Shijiazhuang, 050000 Hebei China; 2 Department of Neurologic Medicine, the Second Hospital of Hebei Medical University, Shijiazhuang, 050000 Hebei China; 3 Department of Medical Oncology, the Second Hospital of Hebei Medical University, Shijiazhuang, 050000 Hebei China; 4 The Public Health College, Hebei Medical University, Shijiazhuang, 050000 Hebei China; 5 Department of Radiology, the Second Hospital of Hebei Medical University, Shijiazhuang, 050000 Hebei China

**Keywords:** Disseminated Cryptococcosis, Metastatic tumor, Transverse myelitis, Immunocompetent, Cryptococcosis pneumonia, Tuberculosis

## Abstract

**Background:**

Transverse myelitis (TM) is due to inflammatory spinal cord injury with bilateral neurologic involvement, which is sensory, motor, or autonomic in nature. It may be associated with autoimmune disease, vaccination, intoxication and infections. The most common infection cause of TM is Coxsackie virus and Mycoplasma pneumoniae. The cryptococcosis is rare. We present the case of disseminated cryptococcosis revealed by transverse myelitis in an immunocompetent 55-year-old male patient. The literature review is also stated.

**Case presentation:**

The 55-year-old man suffered from gradual numbness, weakness in both lower limbs and finally paralyzed in the bed. The thoracic spine Computed tomography (CT) was normal, but multiple nodules in the lung were accidentally discovered. Thoracic Magnetic Resonance Imaging (MRI) showed diffused thoracic spinal cord thickening and extensively intramedullary T2 hyper intensity areas. Gadolinium contrast enhanced T1WI showed an intramedullary circle-enhanced nodule at 9th thoracic level. Diagnosis was made by histological examination of the bilateral lung biopsy. The patient was treated successfully with systemic amphotericin B liposome and fluconazole and intrathecal dexamethasone and amphotericin B liposome.

**Conclusions:**

This is a patient with disseminated cryptococcosis involving the lung, spinal cord and adrenal glands, which is rare in the absence of immunodeficiency.

## Background

Cryptococcosis has been defined as an opportunistic fungal infection because it mainly occurs in immunocompromised populations. Occasionally, it can occur inimmunocompetent hosts. But the cryptococcal infection with involvement of the lung, spine cord, and adrenal glands in a patient with no detectable immuno deficiency is rare according to the literature, and it is often easily misdiagnosed.

## Case presentation

A 55-year-old man, previously healthy, presented to our hospital on Nov 28th, 2017, with a 10-day history of gradual numbness and weakness in both lower limbs. The weakness of the lower extremities gradually developed towards the proximal end, and finally paralyzed the patient in the bed. The patient had urinary incontinence and constipation. He denied injuries to his back, history of herpes zoster or any other opportunistic infections. Physical examination was normal, except in both lower limbs, which had no power in all muscle groups, with hypotonia and hyporeflexia. There was a loss of sensation to pin prick and light touch, up to the level of the tenth thoracic dermatome.

**Fig. 1:**
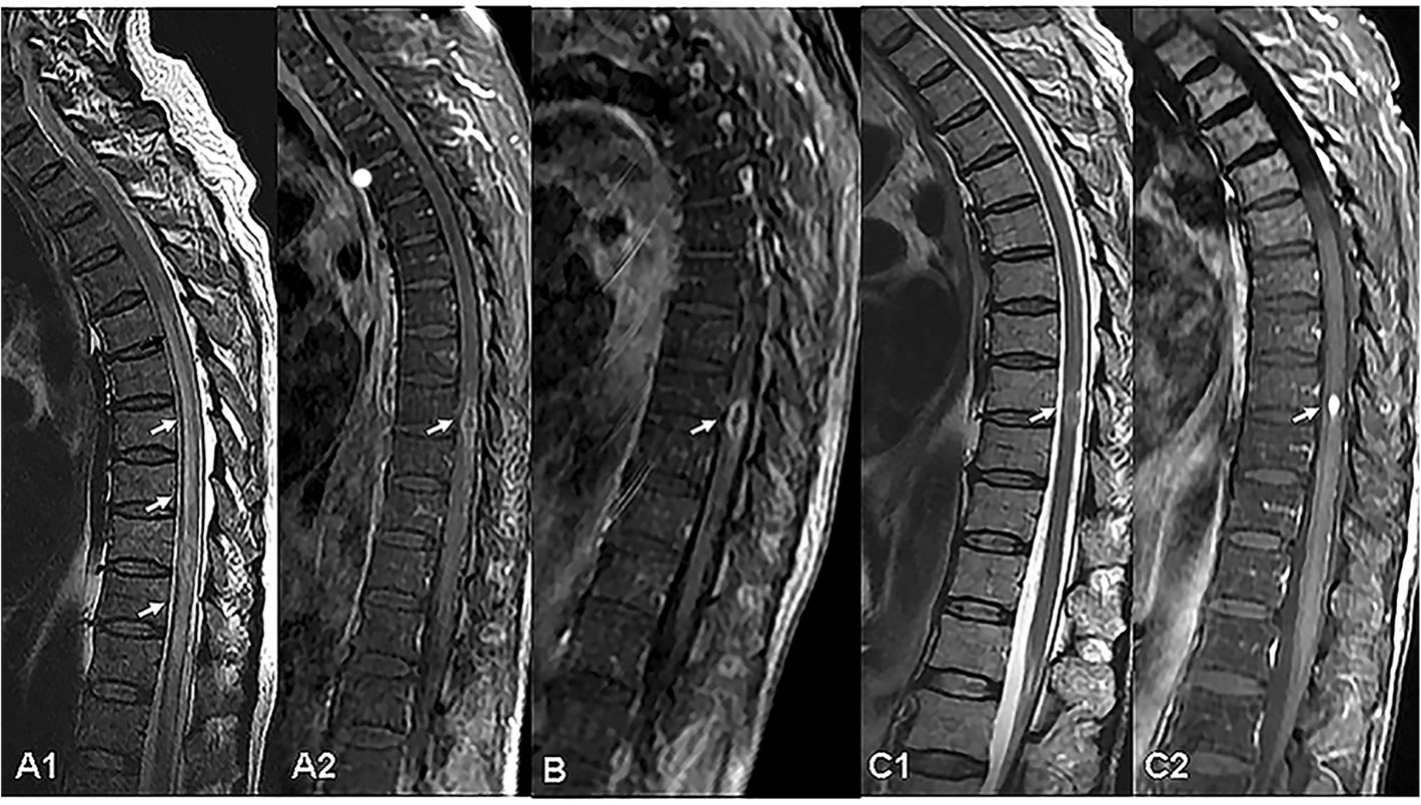
A1 showed longitudinal flaky hyper intense signal in the medullar with a range of approximately 8 vertebrae. A2 showed a small annular enhancement zone in the center of the lesion. B was enhanced T1WI after 2 weeks of treatment, indicating that the intramedullular annular enhancement zone was not significantly reduced. C1-C2 were images of follow up 3 months of oral fluconazole 400 mg/day. C1 showed a intramedullary hyper intense signal patch, with a longitudinal extent less than 1vertebrae.C2 showed a nodular enhancement area, significantly smaller than before

**Fig. 2:**
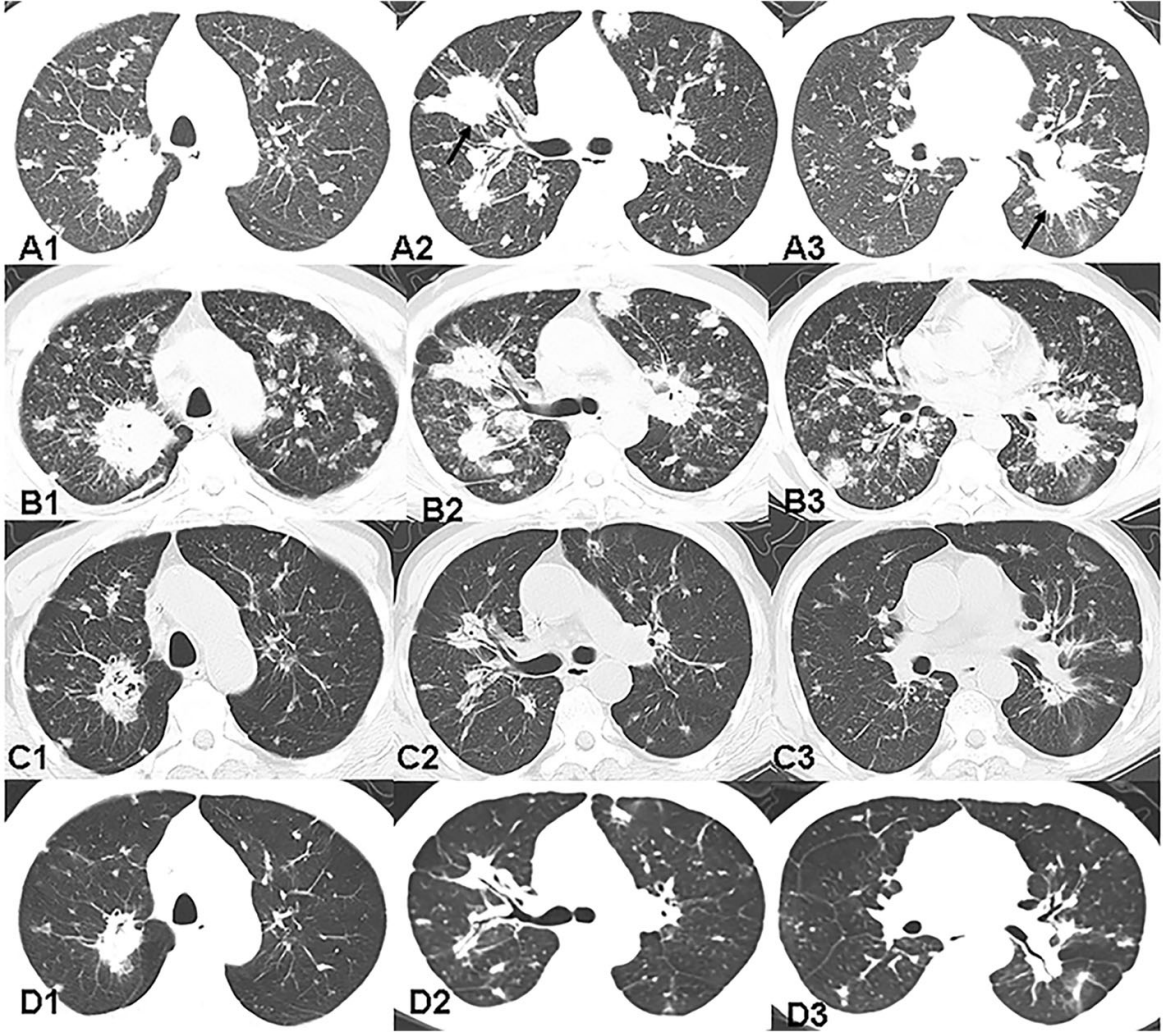
A1-A3 was CT images of lung oftheThoracic Hospital on October 25, 2017, showing multiple spherical lesions in both lungs. Some lesions were closely related to the bronchus (black arrows), and most of the other lesions were randomly distributed. B1-B3 showed the number of lung nodules increased before antifungal treatment. C1-C3 were re-examination after 2 weeks of treatment, showing lesions in the lung significantly reduced. D1-D3 were images follow up 3 months of oral fluconazole 400 mg/d, showing that the lung lesions were further reduced

Laboratory data revealed the C-reactive protein (CRP) level was 166 mg/L, tumor markers such as neuron-specific enolase (NSE) and ferroprotein (FER) were slightly abnormal. The aspergillus galactomannan was 0.90μg/ml. The blood sugar level and T-cell spot test for tuberculosis infection (T-SPOT.TB) were normal. Serological tests were negative for human immunodeficiency virus (HIV), hepatitis B virus (HBV) and autoantibody. MRI of the brain was normal. CT of the thoracic spine was normal, but multiple nodules in the lung were accidentally discovered. Further chest CT examination showed a large number of randomly distributed nodules with varying sizes, unsmooth boundaries and a small number of burrs. (Fig. 2A1-3). One nodule in right adrenal gland was accidentally discovered at the same time. Thoracic MRI showed diffused spinal cord thickening and extensively T2WI hyper intensity areas (Fig. 1A1-A2). Gadolinium contrast enhanced T1WI showed an intramedullary circle-enhanced nodule at 9th thoracic level (Fig. 1B).

A careful history examination revealed that the patient developed cough, expectoration without fever 7 months ago. He didn’t take it seriously. He presented to a local hospital with aggravated cough and expectoration 3 months ago. The chest CT showed multiple nodules, which were considered tumor. For the first time the lung needle biopsy guided by CT was performed in local hospital. The histopathological result was bronchial mucous membrane and lung tissue granulomatous disease with no necrosis; no tumor cells and the possibility of tuberculosis could not be excluded. Then the patient was admitted to a tuberculosis hospital and was given anti-tuberculosis treatment for 1 month. But reexamination of the lung CT showed exacerbation of the diseases (Fig. 2B1-3). He was again admitted to another higher hospital. Because of the multiple nodules of lung CT, in order to further eliminate the possibility of tumor, he had a tracheoscopy and no tumor was found. Ten days ago, he presented to our hospital with weakness in lower limbs, urinary incontinence, and constipation.

**Fig. 3:**
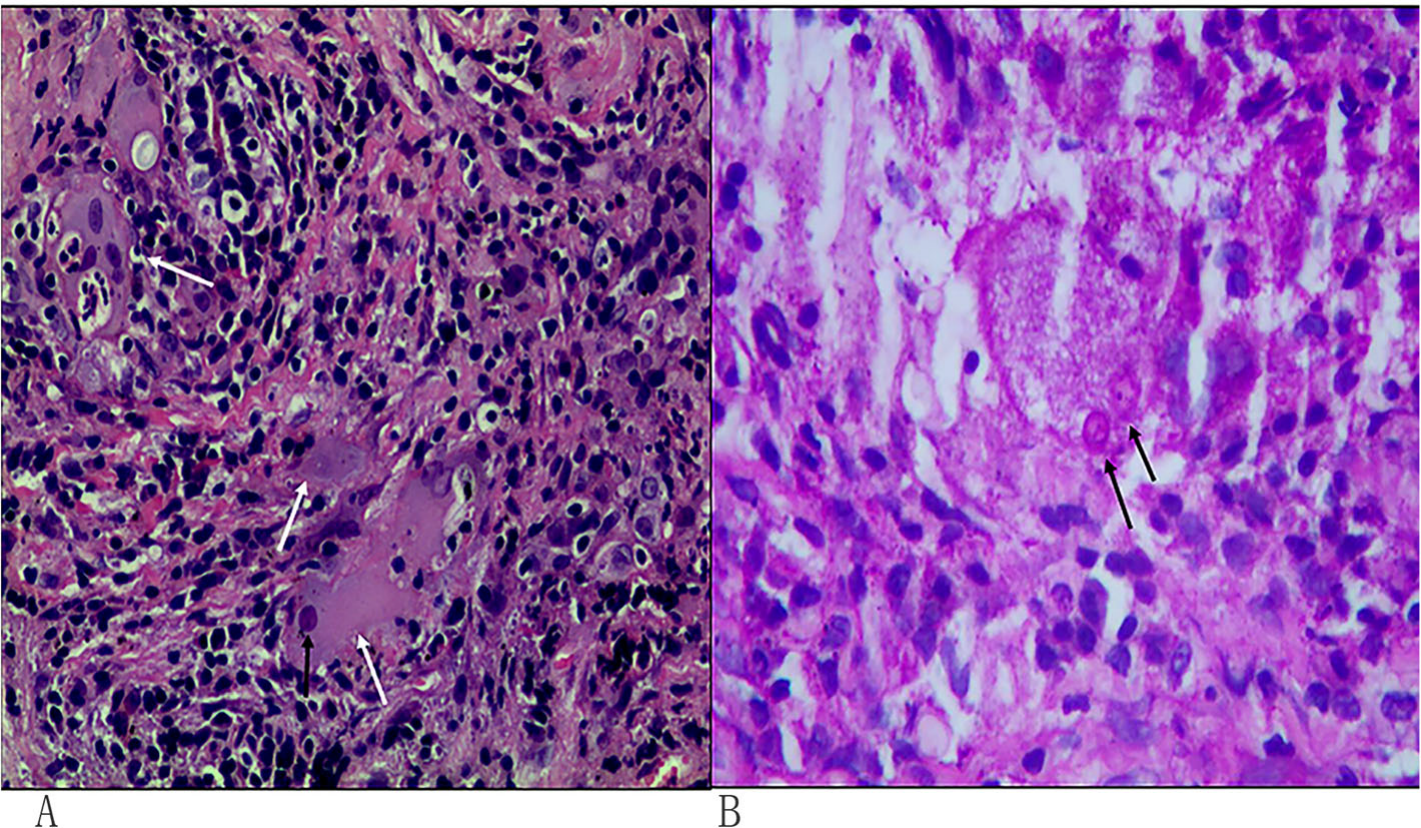
**a** was a HE stain image of lung biopsy material. Macrophage aggregation (white arrow) was seen, which indicate granulomatous lesions. Round microbes (black arrow) within macrophages were suspected fungi. **b** showed positive stained round microbes (black arrow) in macrophages with PAS stain, consistent with Cryptococcus

The needle biopsy in the lesion of right upper lung lobe was performed again. HE staining indicated that the size varying cryptococcus was engulfed by macrophages, which were surrounded by massively infiltrated lymphocytes and a large amount of fibrous tissue that formed granulomas (Fig. 3a). Periodic acid Schiff staining (PAS staining) indicated accumulated cryptococcus spores were located in the endochylema of multinucleated giant cells. The cell wall was stained red. PAS staining showed positive round microbes (Fig. 3b). Cryptococcus infection was diagnosed. The cerebrospinalfluid (CSF) revealed a chlorine level of 117.9 mmol/L and normal white cell count, glucose level and protein level. Abnormal activation of monocytes can be seen in CSF cytology. Cryptococcus India ink stains and cryptococcus specific antigen were all negative.

Questioning to determine exposure to cryptococcus revealed that the patient had exposure to bird droppings. The patient received anti-mycotic therapy with amphotericin B liposome 150 mg daily and fluconazole 400 mg daily for vein. Two weeks later, lung CT showed a shrink of nodules, also the exudation and right adrenal gland nodule reduced (Fig. 2C1-3). Thoracic MRI showed no significant change in intramedullary lesion. One month later, the sensory level of the patient declined slightly and the muscle strength of the lower limbs did not change. He was given intrathecal dexamethasone 5 mg and amphotericin B liposome 0.01 mg one time. One week later, a repeated lumbar puncture revealed that CSF, white cell count, chlorine level, glucose level and protein level were normal. Cryptococcus India ink stains and microbial cultures were all negative. He was transited to oral fluconazole on discharge.

Follow-up 3 months after discharge, thoracic MRI showed that the nodular lesion shrunk and the swelling reduced significantly (Fig. 1C1-2). The lung CT showed the multiple nodules and masses gradually decreased (Fig. 2D1-3). He had no cough, neither fever, and a slight decline in the sensory level and his lower limb muscle power had slight improvement.

## Discussion and conclusions

Cryptococcus is a nonmycelial, budding, encapsulated, opportunistic, pathogenic, and yeast-like fungus. Inhalation of either the yeast form or basidiospores from an environmental source, such as bird droppings or soil, is believed to cause infection with this opportunistic fungus [1]. Cryptococcosis usually occurs in patients with tuberculosis, diabetes, leukemia, lymphoma, organ transplantation, acquired immune deficiency syndrome (AIDS), and histoplasmosis [2]. Occasionally, it can occur in a small subset of immunocompetent hosts, with an incidence estimated at 0.2 per million per year [3]. Cryptococcosis is an invasive fungal disease that invades the central nervous system, lung, skin, lymph nodes, bone marrow, etc., causing chronic inflammation and granuloma. The most frequent disease manifestation is meningoencephalitis. But cryptococcal pneumonia has not been diagnosed because of nonspecific respiratory symptoms. Cryptococcal disease can present as a cough, sputum, shortness of breath, mild fever, chest pain and other symptoms.

The image differentiation between cryptococcosis and malignant tumor is difficult. Multiple pulmonary nodules and/or masses are often mistaken for primary or metastatic malignancy [4]. It is also difficult to distinguish cryptococcal infection from tuberculosis by a surgical specimen, or demonstration in culture. The pus, caseous necrosis, and granulation within cryptococcal lesions are similar to those in tuberculosis. Therefore, observation of these fungal spores, circular mycelium, is essential for a diagnosis of cryptococcal infection [5]. Pathological PAS staining is positive in the tissue specimens of percutaneous pulmonary puncture, which should be differentiated from *Histoplasma capsulatum*.

Thoracic MRI showed diffused spinal cord thickening and extensively T2WI hyper intensity areas. Gadolinium contrast enhanced T1WI showed an intramedullary circle-enhanced nodule at 9th thoracic level. In general, the high signal on T1-weighted MR images may be due to the presence of hemorrhage, melanin, fat, or granulomatous tissue [6]. Combined with lung histopathology, the final diagnosis was invasive cryptococcosis. Although no mycological evidence for cryptococcus was found in the patient’s CSF, after treatment with amphotericin B liposome and fluconazole, spinal cord lesions decreased rapidly from a wide range of T2 hyperintense edematous zones, and presented nodular enhancement, which were consistent with the imaging features of granulomatous lesion.

There are some important lessons from our case. First, Cryptococcus can rarely cause spinal cord infections apart from lung, meninges, and parenchyma. When suspecting systemic infectious disease involving the lungs, brain, meninges or spinal cord, the possibility of cryptococcus infection should be taken into account; Second, the radiological presentation of multiple nodules and masses in lungs, one nodule in right adrenal gland and the spinal cord lesions may easily be misdiagnosed as pulmonary metastases or tuberculosis. The disease is difficult to diagnose and progress gradually, leading to paraplegia. Third, when biopsy tissue pathology revealed granulomatous lesions, not only acid-fast staining to exclude tuberculosis, but also PAS staining to exclude cryptococcosis should be considered.

CSF pleocytosis, elevated CSF IgG index, or abnormal gadolinium enhancement of the spinal cord on MRI is necessary to diagnosis of TM [7]. In our case the patient has abnormal CSF and gadolinium contrast enhanced T1WI showed an intramedullary circle-enhanced nodule at 9th thoracic level, therefore, it can be diagnosed as TM. Cryptococcus rarely causes spinal cord infection, leading to transverse myomyelitis [8]. To our knowledge; six patients with intramedullary cryptococcus have been reported in the literature [8–13]. All patients were immunocompetent. Only the case reported by Scullery [12] had a pulmonary cryptococcal infection 6 months before intramedullary localization, which is consistent with our patient.

With the deepening understanding of cryptococcosis, the diagnosis of cryptococcosis in immunocompetent patients gradually increases. When those immunocompetent patients have pulmonary lesions with meningeal or spinal cord lesions, we should think of disseminated cryptococcosis.

## Data Availability

All relevant data to this case is reported in the manuscript.

## Abbreviations

CNS: Central nervous system
CRP: C-reactive protein
CSF: Cerebrospinal fluid
CT: Computed tomography
MRI: Magnetic resonance imaging
PAS: Periodic Acid-Schiff stain

## References

[1] Mitchell DH, Sorrell TC, Allworth AM, Heath CH, McGregor AR, Papanaoum K, Richards MJ, Gottlieb T (1995). Cryptococcal disease of the CNS in immunocompetent hosts: influence of cryptococcal variety on clinical manifestations and outcome. Clin Infect Dis.

[2] Baddley JW, Perfect JR, Oster RA, Larsen RA, Pankey GA, Henderson H, Haas DW, Kauffman CA, Patel R, Zaas AK (2008). Pulmonary cryptococcosis in patients without HIV infection: factors associated with disseminated disease. Eur J Clin Microbiol Infect Dis.

[3] Friedman GD (1983). The rarity of cryptococcosis in northern California: the 10-year experience of a large defined population. Am J Epidemiol.

[4] Wang C, Jia N, Zhang L, Liu K, Liu H, Yu H (2014). Imaging findings of cryptococcal infection of the thoracic spine. Int J Infect Dis.

[5] Gupta SK, Chhabra R, Sharma BS, Das A, Khosla VK (2003). Vertebral cryptococcosis simulating tuberculosis. Br J Neurosurg.

[6] Farrokh D, Fransen P, Faverly D (2001). MR findings of a primary intramedullary malignant melanoma: case report and literature review. AJNR Am J Neuroradiol.

[7] Krishnan C, Kaplin AI, Pardo CA, Kerr DA, Keswani SC (2006). Demyelinating disorders: Update on transverse myelitis. Curr Neurol Neurosci.

[8] Gültaşli NZ, Ercan K, Orhun S, Albayrak S (2007). MRI findings of intramedullary spinal cryptococcoma. Diagn Interv Radiol (Ankara, Turkey).

[9] Su MC, Ho WL, Chen JH (1994). Intramedullary cryptococcal granuloma of spinal cord: a case report. Zhonghua Yi Xue Za Zhi (Taipei).

[10] Grosse P, Tintelnot K, Sollner O, Schmitz B (2001). Encephalomyelitis due to Cryptococcus neoformans var gattii presenting as spinal tumour: case report and review of the literature. J Neurol Neurosurg Psychiatry.

[11] Ramamurthi B, ANGULI VC (1954). Intramedullary cryptococcic granuloma of the spinal cord. J Neurosurg.

[12] FM S (1961). Cryptococcic granuloma of the dorsal spinal cord. A case report. Neurology.

[13] Lai PH, Wang JS, Chen WL, Pan HB, Yang CF (2001). Intramedullary spinal cryptococcoma: a case report. J Formos Med Assoc.

